# Study on Modeling and Evaluating Alfalfa Yield and Optimal Water Use Efficiency in the Agro-Pastoral Ecotone of Northern China

**DOI:** 10.3390/plants13020229

**Published:** 2024-01-14

**Authors:** Xiangyang Miao, Guoshuai Wang, Ruiping Li, Bing Xu, Hexiang Zheng, Delong Tian, Jun Wang, Jie Ren, Zekun Li, Jie Zhou

**Affiliations:** 1 College of Water Conservancy and Civil Engineering, Inner Mongolia Agricultural University, Hohhot 010018, China; miaoxiangyang1108@163.com (X.M.); zhoujiepiaoliang@163.com (J.Z.); 2 Yinshanbeilu Grassland Eco-Hydrology National Observation and Research Station, China Institute of Water Resources and Hydropower Research, Beijing 100038, China; imau_wgs@163.com (G.W.); mkszhx@163.com (H.Z.); mkstdl@126.com (D.T.); wangjun@iwhr.com (J.W.); renj@iwhr.com (J.R.); mkslzk@163.com (Z.L.); 3 Institute of Water Resources for Pastoral Area Ministry of Water Resources, Hohhot 010020, China

**Keywords:** agro-pastoral ecotone, alfalfa, yield, Dssat-Forages-Alfalfa model, water use

## Abstract

The agro-pastoral ecotone in northern China is the main production area of agriculture and animal husbandry, in which agricultural development relies entirely on groundwater. Due to the increasing water consumption of groundwater year by year, groundwater resources are becoming increasingly scarce. The substantial water demand and low germination rate in the first year are the main characteristics of alfalfa (*Medicago sativa* L.) yield in the agro-pastoral ecotone in northern China. Due to unscientific irrigation, water resources are seriously wasted, which restricts the development of local agriculture and animal husbandry. The study constructed the Dssat-Forages-Alfalfa model and used soil water content, leaf area index, and yield data collected with in situ observation experiments in 2022 and 2023 to calibrate and validate the parameters. The study found ARE < 10%, E_NRMS_ < 15%, and R^2^ ≥ 0.85. The model simulation accuracy was acceptable. The study revealed that the water consumption at the surface soil layer (0–20 cm) was more than 6~12% and 13~31% than that at the 20–40 cm and 40–60 cm soil layers, respectively. The study showed when the irrigation quota was 30 mm, the annual yield of alfalfa (*Medicago sativa* L.) (7435 kg/ha) was consistent with that of the irrigation quota of 33 mm, and increased by 3.99% to 5.34% and 6.86% to 10.67% compared with that of irrigation quotas of 27 mm and 24 mm, respectively. To ensure the germination rate of alfalfa (*Medicago sativa* L.), it is recommended to control the initial soil water content at 0.8 θ*_fc_*~1.0 θ*_fc_*, with an irrigation quota of 30 mm, which was the best scheme for water-use efficiency and economic yield. The study aimed to provide technological support for the rational utilization of groundwater and the scientific improvement of alfalfa yield in the agro-pastoral ecotone in northern China.

## 1. Introduction

In recent years, there has been an increasing global demand for beef, mutton, and dairy products, leading to a pronounced supply gap for forage-fed livestock. A crucial issue is the shortage of high-quality forage. The total demand for high-quality forage in China exceeds 120 million tons. In order to ensure self-sufficiency, a forage gap of 50 million tons depends on imports. As high-quality forage, alfalfa has a huge import volume, accounting for 80% of the total import of grass products. Domestic alfalfa urgently needs to improve yield to make up for the gap. The agro-pastoral ecotone in northern China is an important breeding area for cattle and sheep, as well as a major production area for cultivated forage. Alfalfa, a robust perennial legume known for its rich nutritional profile and high yield potential, thrives despite harsh conditions and intensive grazing, yielding multiple harvests over successive years [1,2,3]. Alfalfa is a prized high-quality forage in the agro-pastoral ecotone with severe land desertification, in which agricultural irrigation relies entirely on groundwater. Due to the overexploitation of groundwater, groundwater resources are becoming increasingly scarce, which restricts the development of agriculture and animal husbandry in northern China [4,5]. Alfalfa’s inherently high water demand means that its cultivation is subject to the vicissitudes of the local climate and the limited irrigation resources [6,7]. Consequently, pioneering research into water-efficient, yield-enhancing techniques for alfalfa is a pressing necessity. Tian et al. [8] undertook a pot experiment that involved imposing various water and salt stress conditions to ascertain their influence on alfalfa’s water consumption, growth, and physiological responses. Their findings revealed an optimal range of soil salinity (2–3 g/kg) and irrigation (70–85% of field capacity) conducive to alfalfa growth, shedding light on how abiotic stressors can modulate crop performance. Tong et al. [9] conducted a two-year field irrigation study to examine the relationship between soil moisture gradients under drip irrigation and key agronomic parameters such as the crop coefficient (Kc) and water use efficiency (WUE). Ma et al. [10] embarked on comparative experiments to discern the most effective irrigation technique for alfalfa in the arid and semi-arid regions of northwest China. By evaluating methods such as rolling irrigation, sprinkler irrigation, and subsurface drip irrigation, they determined that subsurface drip irrigation held the most promise. It markedly improved soil moisture profiles, boosted alfalfa yields, and enhanced the overall efficiency of water use in irrigation practices. Zheng et al. [11] employed a water balance approach and FAO-56 guidelines to ascertain the evapotranspiration rates and the optimal amount and depth of subsurface drip irrigation, concluding values of 22.5–30.0 mm and 20 cm, respectively. In a similar quest, Wang Xing et al. [12] utilized a two-factor split-plot design to determine the ideal irrigation and fertilization rates for alfalfa seed production in Ningxia’s drip-irrigated fields. These contributions have significantly enhanced the alfalfa cultivation knowledge base. However, there is a noted deficiency in research addressing the unique conditions of agro-pastoral ecotones with sloping terrain, shorter growth windows, and limited thermal accumulation, underscoring the need for tailored agricultural practices in these distinct environments. Furthermore, many scholars have studied alfalfa yield and irrigation schemes, but there has been less consideration of the interplay among water, soil, and crops. Currently, turning green is critical for alfalfa survival, and initial soil water content is a key factor for turning green in alfalfa. However, the optimal initial soil water content for alfalfa has not yet been identified.

As technology has evolved, the application of computers in agriculture has become increasingly widespread. Scholars have undertaken extensive research on crop growth models, among which the Decision Support System for Agrotechnology Transfer (DSSAT) is one of the most widely applied models globally [13,14]. The DSSAT model is primarily used for agricultural yield forecasting, experimental analysis, and the study of climate impacts on agricultural production [15,16,17]. Currently, the model is capable of simulating the growth of 26 different crops, including wheat, corn, potatoes, et al. Wang Bin et al. [18] demonstrated the integration of Particle Swarm Optimization (PSO) with the Decision Support System for Agrotechnology Transfer (DSSAT), offering an innovative approach to refine the calibration of rice variety parameters. Their validation through comparative analysis underscores the potential of PSO to enhance crop modeling accuracy. Zhu et al. [19] incorporated canopy remote sensing data, using Leaf Area Index (LAI) and Leaf Nitrogen Accumulation (LNA) as proxy variables. This novel method provided insights into the estimation of grain protein content and overall yield for corn. Wan et al. [20] harnessed the DSSAT model to inform and improve irrigation strategies for summer maize, introducing a method to guide irrigation timing by comparing cumulative rainfall with cumulative evapotranspiration (ETc) data. Song et al. [21] focused on refining the CERES-Maize model’s capacity to simulate maize growth under water-limited conditions. Their work not only addressed the effects of drought on summer maize but also considered the broader implications of climate change, such as rising temperatures and shifts in maize planting regions, on future crop yields. Xiang et al. [22] leveraged the coupled DSSAT-Modflow modeling system, implementing sensitivity analysis to pinpoint key environmental and management variables affecting groundwater storage and crop yield, thereby facilitating informed decisions in groundwater-reliant agricultural zones. Shen et al. [23] developed a novel methodology to estimate soil evaporation for fields with plastic film mulching, integrating this advancement into the DSSAT framework and enhancing the model’s comprehensiveness. Qu et al. [24] utilized the DSSAT-CERES-Wheat model to simulate winter wheat yield responses to climate variability and projected CO_2_ concentration pathways (PCR4.5 and PCR8.5), shedding light on the potential adversities posed by increased thermal resources under varying meteorological scenarios. These studies corroborate the efficacy of the DSSAT model in accurately simulating crop growth and yield across diverse conditions, showcasing its robust applicability in various contexts. Nevertheless, in northern China’s arid regions, current research on alfalfa irrigation quota optimization and methods remains anchored in field experimentation, with limited incorporation of such advanced modeling techniques. Field irrigation experiments are subject to many influencing factors, entail considerable labor and resource consumption, and suffer from inconsistent monitoring standards, leading to considerable variability in results within the same region. In the future, it will be necessary to combine field experiments with the DSSAT model to reduce the human and material resources required for field experiments. By optimizing alfalfa variety parameters, the applicability and accuracy of the DSSAT model can be enhanced.

Therefore, this study calibrated and validated the Dssat-Forages-Alfalfa model to simulate the growth process of alfalfa during the growing season and the soil water content dynamics in the agro-pastoral ecotone. This research delves into the interplay between varying irrigation quotas and the initial soil water content on alfalfa yield. It is premised on the dual objectives of maximizing water resource efficiency and achieving economic gains for local agricultural systems. It will determine the precise water needs of alfalfa at different growth stages under the unique climatic conditions of Inner Mongolia, thereby minimizing over-irrigation and conserving water resources. It will also establish irrigation schedules based on the soil water content, ensuring water is supplied when it is most beneficial to the crop and reducing unnecessary water application. This study aimed to provide a theoretical basis for the efficient utilization of groundwater and the scientific enhancement of alfalfa yield in the agro-pastoral ecotone, Inner Mongolia.

## 2. Results

### 2.1. Model Calibration and Validation

This study used experimental data (soil water content, yield, and leaf area index) collected in 2022 and 2023 to calibrate and validate ten genetic parameters using the trial and error method [25]. As shown in Table 1, the calibration accuracy for leaf area under different irrigation quota treatments was indicated by ARE values ranging from 5.34% to 7.67%, E_NRMS_ from 9.38% to 13.47%, and R^2^ from 0.85 to 0.92; for yield under different irrigation treatments, the calibration accuracy was reflected by ARE values ranging from 3.77% to 5.87%, E_NRMS_ from 9.26% to 10.74%, and R^2^ from 0.89 to 0.93. For soil water content under different irrigation treatments, the calibration accuracy was ARE ≤ 6%, E_NRMS_ ≤ 15%, and R^2^ ≥ 0.85 (Table 2). All calibrated genetic parameters were set into the model for validation. The validation results are shown in Table 1 and Table 2. The ARE values of LAI and yield ranged from 1.87% to 8.11%, E_NRMS_ ranged from 3.47% to 12.88%, and R^2^ ranged from 0.87 to 0.96. For soil water content in 0–60 cm soil layers, the ARE value ranged from 1.01% to 5.04%, E_NRMS_ from 8.49% to 13.73%, and R^2^ from 0.87 to 0.95. It showed a good agreement between simulated and observed values for soil water content, leaf area index, and yield. The calibrated and validated genetic parameters are shown in Table 3. The study showed that the Dssat-Forages-Alfalfa model can accurately capture the essential process of the crop’s development under differing irrigation scenarios.

### 2.2. Effects of Irrigation on Soil Water Content, Leaf Area Index, and Yield

#### 2.2.1. Effects of Different Irrigation Gradients on Soil Water Content

**Figure 1:**
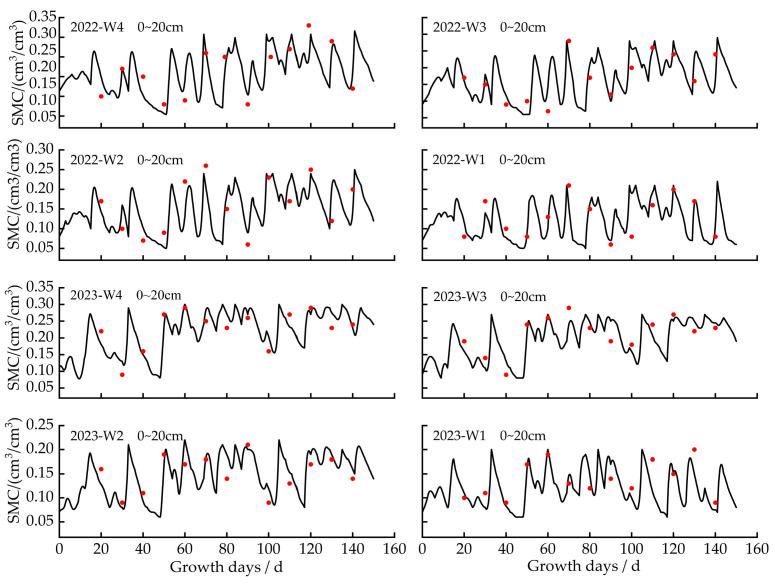
Simulated and measured values of soil moisture content (SMC) in the 0–20 cm soil layer with different treatments in 2022 and 2023. Note: The red dot is the measured value, and the solid line is the simulated value.

**Figure 2:**
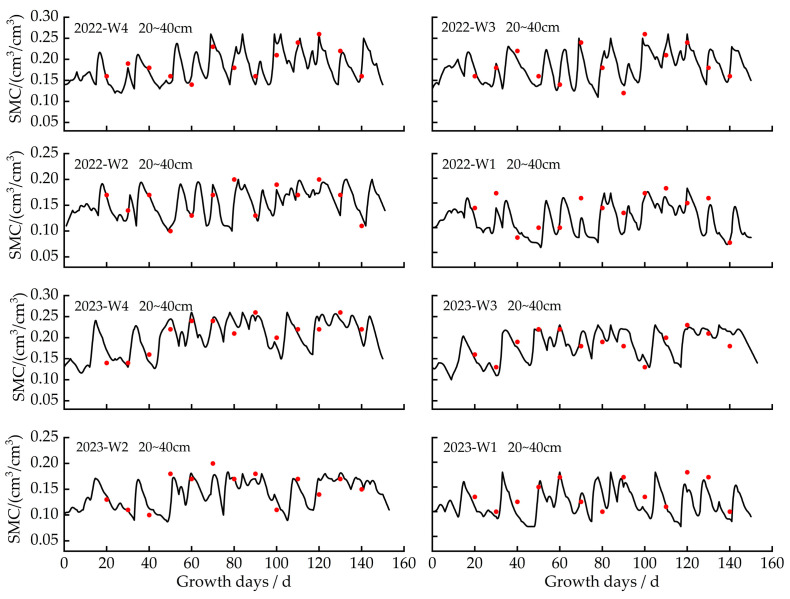
Simulated and measured values of soil moisture content (SMC) in the 20–40 cm soil layer with different treatments in 2022 and 2023.Note: The red dot is the measured value, and the solid line is the simulated value.

**Figure 3:**
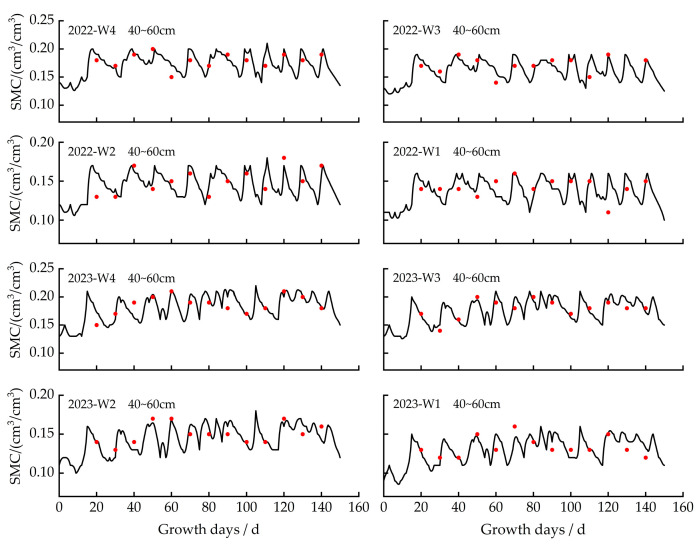
Simulated and measured values of soil moisture content (SMC) in the 40–60 cm soil layer with different treatments in 2022 and 2023. Note: The red dot is the measured value, and the solid line is the simulated value.

Since the soil layer below 60 cm was predominantly composed of small gravel, the cultivated soil layer is notably thin (0–20 cm). The study mainly focused on the soil water content in the 0–60 cm soil layer. Soil water dynamics in the 0–60 cm soil layer under different irrigation quotas were depicted in Figure 1, Figure 2 and Figure 3. In 2022 and 2023, the peak soil water content for the 0–20 cm, 20–40 cm, and 40–60 cm soil layers was 0.31 cm^3^/cm^3^, 0.26 cm^3^/cm^3^, and 0.22 cm^3^/cm^3^.

In 2022, soil water content in the 0–20 cm, 20–40 cm, and 40–60 cm soil layers varied from 0.05 cm^3^/cm^3^ to 0.31 cm^3^/cm^3^, from 0.06 cm^3^/cm^3^ to 0.26 cm^3^/cm^3^, and from 0.10 cm^3^/cm^3^ to 0.21 cm^3^/cm^3^. In 2023, for different irrigation quotas, the soil water content ranged from 0.06 cm^3^/cm^3^ to 0.30 cm^3^/cm^3^ for the 0–20 cm soil layer, 0.07 cm^3^/cm^3^ to 0.26 cm^3^/cm^3^ for the 20–40 cm soil layer, and 0.08 cm^3^/cm^3^ to 0.22 cm^3^/cm^3^ for the 40–60 cm soil layer. The study found that soil water retention and storage capacity were poor. After each irrigation, soil water content declined rapidly.

After each irrigation to the next irrigation event in 2022 and 2023, soil water in the 0–20 cm soil layer reduced by around 37–83%, in the 20–40 cm soil layer, it decreased by approximately 28–77%, and in the 40–60 cm soil layer, it reduced by 24–64%. The water consumption in the 0–20 cm soil layer was 6–12% and 13–31% higher than that in the 20–40 cm and 40–60 cm soil layers, respectively.

#### 2.2.2. Effects of Different Irrigation Amounts on Alfalfa Leaf Area

**Figure 4:**
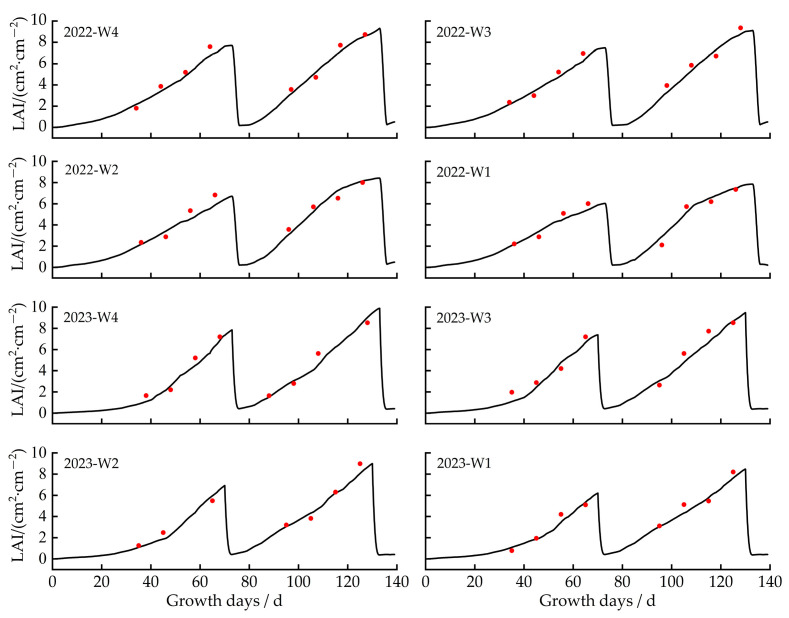
Simulated and measured leaf area index (LAI) values for different treatments in 2022 and 2023. Note: The red dot is the measured value, and the solid line is the simulated value.

The changes in leaf area index (LAI) for alfalfa under various irrigation gradients between 2022 and 2023 are depicted in Figure 4. Owing to the lower temperature in the agro-pastoral ecotone, alfalfa had a relatively short growing period. Alfalfa is harvested two times during the growth period every year. Therefore, after each harvest of alfalfa, its leaf area becomes zero, and then with the growth of alfalfa, the leaf area gradually increases. In 2022, the peak values of the LAI under the 33 mm, 30 mm, 27 mm, and 24 mm irrigation quotas were 7.23–8.86, 7.06–8.43, 6.07–7.86, and 5.53–7.31, respectively. In 2023, the peak values of the LAI under the 33 mm, 30 mm, 27 mm, and 24 mm irrigation quotas were 7.85–9.88, 7.38–9.48, 6.92–8.99, and 6.20–8.46, respectively. In 2022 and 2023, the LAI reached the peak value under the 33 mm irrigation quota, more 14–21% than that under the 24 mm irrigation quota. The study found that leaf area in 2023 was larger than that in 2022. The value of LAI of the first harvest in 2023 was 7.90% higher than that of the first harvest in 2022, and the second harvest in 2023 was 10.32% higher than that of the second harvest in 2022.

#### 2.2.3. Effects of Different Irrigation Gradients on Alfalfa Yield

**Figure 5:**
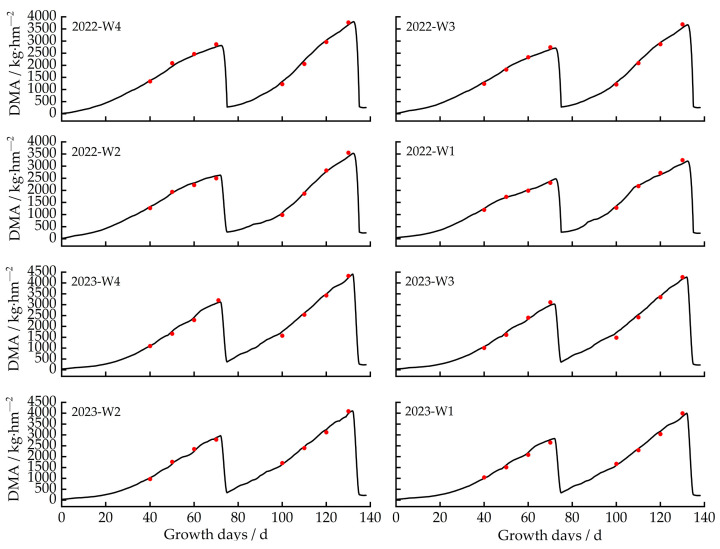
Simulated and measured values of dry matter accumulation under different treatments in 2022 and 2023. Note: The red dot is the measured value, and the solid line is the simulated value.

The dynamics of dry matter accumulation under various irrigation gradients between 2022 and 2023 for alfalfa are depicted in Figure 5. When the irrigation quota for alfalfa in 2022 was 33 mm, 30 mm, 27 mm, and 24 mm, the dry matter mass of the first harvest was 2878 kg·hm^−2^, 2767 kg·hm^−2^, 2628 kg·hm^−2^, and 2555 kg·hm^−2^, and the second harvest was 3898 kg·hm^−2^, 3763 kg·hm^−2^, 3553 kg·hm^−2^, and 3278 kg·hm^−2^. When the irrigation quota for alfalfa in 2023 was 33 mm, 30 mm, 27 mm, and 24 mm, the dry matter mass of the first harvest was 3157 kg·hm^−2^, 3072 kg·hm^−2^, 2989 kg·hm^−2^, and 2867 kg·hm^−2^, and the second harvest was 4467 kg·hm^−2^, 4363 kg·hm^−2^, 4149 kg·hm^−2^, and 4058 kg·hm^−2^. The yield of the second harvest was 800~1300 kg/ha greater than that of the first harvest.

Due to the multi-year growth features of alfalfa, with the development of the root system and increasing nutrient absorption rate year by year, the study found that dry matter yields in 2023 were higher than those in 2022. Each harvested yield of alfalfa in 2023 under the 33 mm, 30 mm, 27 mm, and 24 mm irrigation quotas were 8.84–12.74%, 9.93–13.75%, 12.08–14.36%, and 10.88–19.22% more than that in 2022. The annual dry matter mass of each harvest with the 30 mm irrigation quota was similar to that of the 33 mm irrigation quota, which was higher by 3.99–5.34% and 6.86–10.67% than that of the 27 mm and 24 mm irrigation quotas, respectively.

### 2.3. Dssat-Forage-Alfalfa Model Simulation Application

#### 2.3.1. Different Simulated Scenario Settings

Initial soil moisture content was an essential factor that influences seed emergence, growth, and yield [26]. Purple alfalfa requires suitable soil moisture to turn green and grow [27]. Therefore, the study used a calibrated Dssat-Forages-Alfalfa model to predict alfalfa yields under different initial soil moisture and irrigation gradients. The study aimed to identify the optimal initial soil moisture content for alfalfa turning green and provide a theoretical basis for enhancing alfalfa yields under appropriate soil water content. Six initial moisture contents were set in the simulation scenario; namely, excess level (1.2 θ*_fc_*, 1.1 θ*_fc_*), normal level (1.0 θ*_fc_*, 0.9 θ*_fc_*), and deficit Level (0.8 θ*_fc_*, 0.7 θ*_fc_*)(θ*_fc_* is the field moisture content), exploring the changes in alfalfa yield at different levels. Four irrigation quotas were set for each simulation scenario, with irrigation quotas of 24 mm, 27 mm, 30 mm, and 33 mm. The irrigation time during the growth period was determined based on alfalfa experiments in 2022 and 2023 (Table 4). There, initial soil moisture scenarios were established: excess levels (1.2 θ*_fc_*, 1.1 θ*_fc_*), normal levels (1.0 θ*_fc_*, 0.9 θ*_fc_*), and deficit levels (0.8 θ*_fc_*, 0.7 θ*_fc_*), where θ*_fc_* is field capacity. Each scenario set the four irrigation quotas for 24 mm, 27 mm, 30 mm, and 33 mm. The irrigation management data in the model during the growth period used monitoring experimental data collected in 2022 and 2023 (Table 4). These scenarios explored how alfalfa yields responded to different soil moisture contents and irrigation scheduling.

#### 2.3.2. Effects of Different Initial Soil Moisture Contents and Irrigation Quotas on Alfalfa Yield

**Figure 6:**
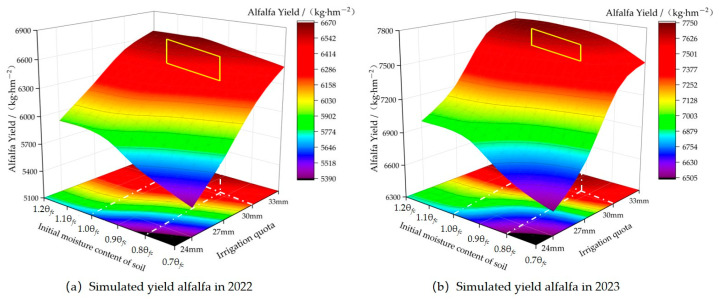
Simulated yield of alfalfa under different treatments in 2022 and 2023. Note: The white dotted line is the optimal irrigation quota and initial soil moisture for local alfalfa, and the yellow box is the optimal yield range.

The Dssat-Forages-Alfalfa model was applied to simulate alfalfa yield under different initial soil moisture contents and irrigation quotas. As shown in Figure 6, when the irrigation quota was 33 mm and the initial soil moisture content was 1.0 θ*_fc_*, the yield was the same as the initial soil moisture content of 1.2 θ*_fc_* and 1.1 θ*_fc_*. However, when the initial soil moisture content was 0.9 θ*_fc_*, 0.8 θ*_fc_*, or 0.7 θ*_fc_*, it is 0.67~0.96%, 1.55~1.86%, and 2.71~3.42% higher, with a relatively small increase in yield. However, the yield was slightly higher at 0.67~0.96%, 1.55~1.86%, and 2.71~3.42% than that at initial soil moisture of 0.9 θ*_fc_*, 0.8 θ*_fc_*, and 0.7 θ*_fc_*, respectively.

When the irrigation quota was 30 mm, the yield at an initial moisture of 1.2 θ*_fc_* and 1.1 θ*_fc_* was similar to that at 1.0 θ*_fc_*. The yield at an initial moisture of 1.0 θ*_fc_* was higher by 0.91~1.97%, 1.89~3.27%, and 3.57~4.96% than that at the initial soil moisture content of 0.9 θ*_fc_*, 0.8 θ*_fc_*, and 0.7 θ*_fc_*, respectively.

When the irrigation quota was 27 mm, the yield at an initial soil moisture of 1.0 θ*_fc_* was 1.86% and 1.71% less than that at initial soil moisture of 1.2 θ*_fc_* and 1.1 θ*_fc_*, while being 3.16~4.94%, 5.86~6.62%, 7.65~8.74% more than that at the initial soil moisture of 0.9 θ*_fc_*, 0.8 θ*_fc_*, and 0.7 θ*_fc_*.

When the irrigation quota was 24 mm, the yield at initial soil moisture of 1.0 θ*_fc_* was 2.53% and 2.29% less than that at initial soil moisture of 1.2 θ*_fc_* and 1.1 θ*_fc_*, while being 4.78~5.34%, 6.25~7.68%, 8.01~9.72% more than that at initial soil moisture of 0.9 θ*_fc_*, 0.8 θ*_fc_*, and 0.7 θ*_fc_*.

With the irrigation amount decreased, the impact of initial soil moisture content on yield gradually became remarkable, and the decrease of yield with high initial soil moisture content was relatively small, while the decrease of yield with low initial soil moisture content was large. The study determined that the best initial soil moisture ranged from 0.7 θ*_fc_* to 1.0 θ*_fc_* for the irrigation quota of 33 mm, from 0.8 θ*_fc_* to 1.0 θ*_fc_* for the irrigation quota of 30 mm, from 0.9 θ*_fc_* to 1.0 θ*_fc_* for the irrigation quota of 27 mm, and from 1.0 θ*_fc_* to 1.1 θ*_fc_* for the irrigation quota of 24 mm. To ensure the maximization of alfalfa turning green rate, efficient utilization of water resources, and economic benefits, we recommended that controlling the initial soil water content to 0.8 θ*_fc_*~1.0 θ*_fc_* with an irrigation quota of 30 mm was the optimal choice.

## 3. Discussion

### 3.1. Effects of Different Irrigation Gradients on Soil Moisture, Leaf Area, and Yield

In this paper, experiments on the water use efficiency and yield of purple alfalfa (cut twice annually) were conducted in the agro-pastoral ecotone of Yinshanbeilu in 2022 and 2023. Soil moisture content, leaf area index, and yield data were used to calibrate and validate the Dssat-Forages-Alfalfa model. The results of the corrected model were in good agreement with the observed data, and they captured the trend of actual alfalfa growth well and effectively. Research by Zhang Song [28] indicated that the DSSAT model provided precise simulations of the effects of different irrigation schemes on the final yield of purple alfalfa, with a Normalized Root Mean Square Error (RMSE) below 0.1. Hou Chen li’s study [29] also suggested that the DSSAT model accurately simulated yields of purple alfalfa under various water and nutrient conditions, with an R^2^ of 0.90. The higher R^2^ in this study compared to Hou Chen li’s could be attributed to the inclusion of fertilizer effects in Hou’s study. Our findings revealed substantial variations in soil moisture across different soil depths. Following each irrigation to the next irrigation in 2022 and 2023, the reduction in surface soil moisture (0~20 cm) exceeded that in deeper layers (20~60 cm) by 6%, 21%, and 7%, 16% respectively. Research by Wu jia Bin et al. [30] revealed that alfalfa root water absorption primarily occurred at the 0~30 cm layer pre-irrigation and the 15–40 cm layer post-irrigation, with more significant fluctuations in moisture in the shallow layers, which aligned well with our study. Conversely, Tian Delong’s research [31] observed more active moisture content changes in deeper soil layers, which could have been due to a shallow water table and deep alfalfa root systems that frequently tapped into groundwater. On the other hand, leaf area increased as irrigation volumes rose, reaching its maximum at the 33 mm irrigation volume for each harvest in 2022 and 2023, and the leaf area at the 33 mm irrigation volume was 24% and 17% and 21% and 14% higher than at the minimal 24 mm volume, respectively. Meng et al. [32] indicated that with consistent light radiation, areas under higher irrigation levels had more extensive leaf coverage than those under lower levels, underscoring that the amount of irrigation water influenced leaf area growth.

### 3.2. Alfalfa Irrigation Scheduling and Yield Prediction Analysis

In this paper, the DSSAT model was used to simulate the effects of varying irrigation quotas on the yield of purple alfalfa. According to the simulation outcomes, the cumulative yield for both harvests at a 30 mm irrigation quota was only modestly reduced by 3.63% and 2.48% compared to the 33 mm quota in 2022 and 2023, respectively. However, they were 5.34% and 3.99% and 10.67% and 6.86% more than that at 27 mm and 24 mm quotas, respectively. Synthesizing the test results and the above analysis, an irrigation schedule of ten events throughout the entire growth cycle of alfalfa with a total water volume of 300 mm was proposed. Zhang Song [27] previously suggested an irrigation volume of 245 mm throughout the alfalfa growth period with 11 irrigation events, targeting water savings, while Tong Chang fu [33] recommended an irrigation volume of 250 mm with 10 events throughout the growth cycle. Our study’s irrigation volumes were higher than those, which might have been due to the shallow soil layers in the research area, an increasing presence of small rocks with depth, and a relative lack of soil water retention and storage capacity. To achieve the soil moisture environment necessary for the growth of purple alfalfa, it was rational to increase the irrigation volume in this area. This study conducted simulations of purple alfalfa yield under various initial soil moisture conditions and irrigation quotas using the verified Dssat-Forages-Alfalfa model. Under the considerations of purple alfalfa’s growth requirements, efficient water resource use, and economic profitability, it was advisable to ensure initial soil moisture levels between 0.8 θ*_fc_* and 1.0 θ*_fc_* before regreening, with an optimal irrigation quota of 30 mm for subsequent growth phases. Zheng et al.’s [11] research suggested an optimized irrigation amount of 22.5 to 30.0 mm per event, with events occurring every 5 to 7 days for alfalfa in their study area. Our study results were consistent with Zheng’s findings. On the other hand, our study identified an optimal alfalfa yield under ideal groundwater utilization conditions at 7435 kg/ha, whereas Zheng had indicated total yields ranging from 7500 to 12,000 kg/ha. The lesser yield in our study was due to factors such as the shorter growing season (100 days) in the agro-pastoral ecotone of Yinshanbeilu, poor soil quality, lack of groundwater recharge, significant diurnal temperature differences, and insufficient heat accumulation. While our region typically had two alfalfa harvests annually, Zheng’s research area in Ordos yielded three harvests per year, resulting in higher production.

This research focused exclusively on simulating purple alfalfa yield under various irrigation quotas, without considering varying fertilizer application strategies. Subsequent studies would employ the Dssat-Forages-Alfalfa model to explore the effects of different water and fertilizer conditions on yields in the agro-pastoral ecotone of Yinshanbeilu.

## 4. Materials and Methods

### 4.1. The Study Area

**Figure 7:**
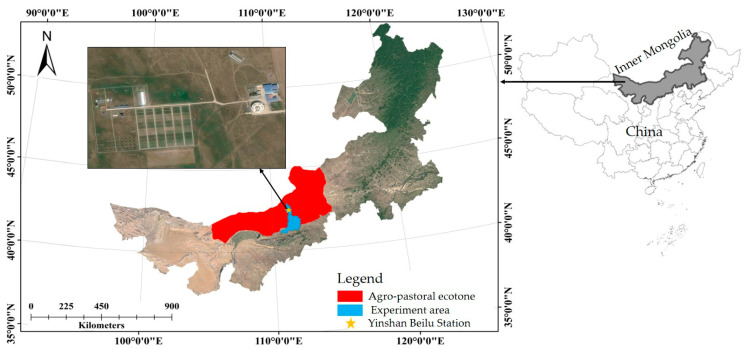
Overview of the study area.

The study area was located in the Yinshanbeilu Grassland Eco-Hydrology National Observation and Research Station (111.209754705° E, 41.351111562° N, elevation 1603 m), which belongs to the agro-pastoral ecotone, Inner Mongolia. The local climate is temperate semi-arid continental monsoon. It is dry and windy in spring and autumn, hot in summer, cold and dry in winter. The average annual precipitation is 284 mm, mostly occurring in July and August, accounting for 80% of the total precipitation. The average annual evaporation is 2305.0 mm. The average annual temperature is about 3.2 °C. The frost-free period lasts an average of 83 days per year, with a maximum of 128 days and a minimum of 58 days. The main crops are potatoes, oats, and alfalfa, and the soil texture is sandy loam soil. As shown in Figure 7.

### 4.2. Field Management and Experimental Design

**Figure 8:**
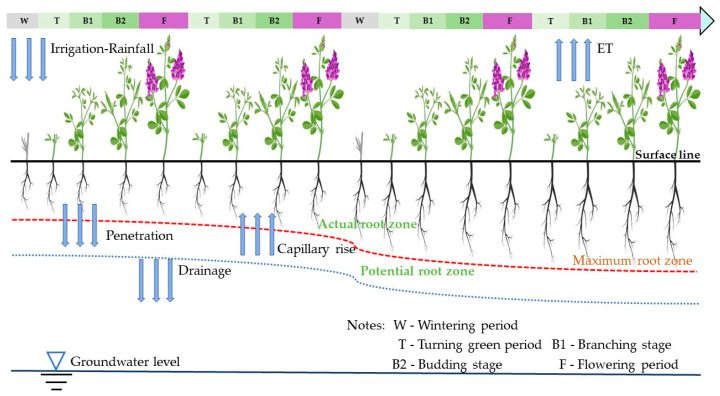
Growth process of alfalfa.

The tested alfalfa variety is “Grassland No.2”. In 2022, Alfalfa went into turning green on 13 May, branched on 3 June, budded on 26 June, flowered on 10 July, and harvested on 25 July and 26 September. In 2023, Alfalfa went into turning green on 12 May, branched on 2 June, budded on 27 June, flowered on 13 July, and harvested on 27 July and 28 September. Alfalfa undergoes the overwintering period starting from early October to May of the following year, which was the shorter growth period. Figure 8 depicts the whole growth process.

The row spacing of alfalfa was 30 cm, and the row spacing of drip irrigation tapes was 30 cm. A drip irrigation tape controlled two rows of alfalfa. The irrigation quota was set at W1 = 33 mm, W2 = 30 mm, W3 = 27 mm, and W4 = 24 mm. Three replicates were set for each treatment, with a total of 12 fields selected randomly. Daily moisture monitoring was performed using TDR (Fleb-30c, Weitu, Inc., China), and irrigation was conducted as soon as the θ*_fc_* was below 50%. Table 4 shows the alfalfa irrigation scheme in the whole growth stage. The experiment selected drip irrigation tape with a diameter of 16 mm, a spacing of 30 cm between drip heads, a flow rate of 2.4 L/h, and a water supply pressure of 0.1 MPa. The experimental field has dimensions of 17 m in length and 8 m in breadth. The irrigation amount of each experimental field was measured and controlled by a water meter installed in the branch pipe. The fertilization during the crop growth period was done manually, with 4 fertilization cycles of 150 kg·hm^−2^ each time, as shown in Table 5.

### 4.3. Data Collection and Methods

#### 4.3.1. Meteorological Data

**Figure 9:**
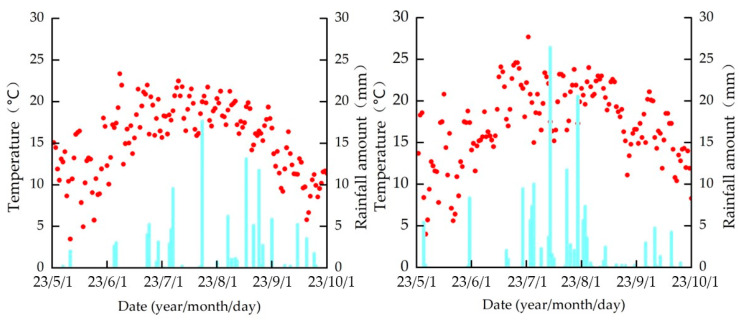
Mean temperature and rainfall during the growth period in 2022 and 2023.

The HOBO-U30 weather station (Onset Computer Corp., Bourne, MA, USA) at the experimental site provided the meteorological data. Figure 9 shows the average temperature and precipitation during 2022–2023. During the growth period in 2022 and 2023, the temperature ranged from 3.5 to 23.5 °C and 3.8 to 27.7 °C, and precipitation was 141.9 mm and 217.3 mm, respectively.

#### 4.3.2. Soil Data

The soil data required for this experiment mainly came from field experiments. These measurements concentrated on the effective root zone, which is divided into five layers: 0–20 cm, 20–40 cm, 40–60 cm, 60–80 cm, and 80–100 cm. The cutting ring method was used to measure the bulk density, saturated water content, and field capacity. High-speed centrifugation was used to calculate the wilting coefficient. Table 6 shows the specific physical properties of the soil.

#### 4.3.3. Alfalfa Field Management

Field management of alfalfa mainly included dates of each growth stage, planting density, fertilizer application amounts, irrigation dates, and irrigation volumes. The field management data of this study were obtained from planting experiments in 2022 and 2023.

#### 4.3.4. Alfalfa Physiological and Ecological Indicators and Yield Determination

The study monitored many indicators, such as soil water content, leaf area index, yield, and phenological period. Five representative alfalfa plants in each field are selected and measured every 10 days. The leaf length L and maximum width Wmax are recorded, and the leaf area of each plant (0.75 LWmax) is calculated. The individual leaf area is accumulated to obtain the leaf area index, based on the planting density of alfalfa. The entire mass of the alfalfa was taken from the branching stage, with three plants in each field. The fresh weight of the plants was then weighed, and after 30 min at 105 °C to kill the green; the plants were dried at 65 °C to a consistent weight, and the weight was measured every fifteen days. Three 1 m × 1 m sample squares were randomly selected from each field to cut alfalfa, leaving 5 cm of stubble, when the alfalfa flowering stage reached 10%. The fresh weight was measured, and it was placed in a 105 °C drying oven for 30 min to kill the green. Then, it was dried at 65 °C to a constant weight, and the dry weight was measured [34,35].

#### 4.3.5. Soil Water Content Monitoring

TDR monitoring equipment was put in each experimental field to track soil moisture dynamics. The study planned to collect soil water data at depths of 10, 20, 40, 60, and 80 cm every 24 h. Soil water content during each growth stage of alfalfa before and after irrigation was collected intensively. At the same time, soil water content monitored by TDR was calibrated.

### 4.4. Crop Growth Model

Crop growth models provide quantitative expressions for the growth process, yield, and the impact of environmental factors on crops [36]. The Decision Supper System for Agricultural Technology Transfer (DSSAT) is one of the most widely used crop growth models in the world, which has attributes like features such as multifunctionality, spatialization, digitization, and visualization; thereby, the DSSAT model can better achieve prediction of crop growth [37].

#### 4.4.1. Soil Water Transport Equation

The formula for calculating changes in soil water content can be expressed as:

(1) ΔS=P+I−EP−ES−R−D

where ∆S is the change of soil water content; P is rainfall; I is the irrigation amount; EP is transpiration; ES is soil evaporation; R is surface runoff; and D is soil profile drainage.

The formula for calculating water stress is as follows:

(2) $$\mathrm{SWD}F_{1}=\frac{WS_{P}}{\mathrm{RWUEP}\times EP_{0}}$$

(3) $$\mathrm{SWD}F_{2}=\frac{WS_{P}}{EP_{0}}$$

where SWDF_1_ is the first water stress factor in the model; WS_P_ is potential root water absorption (mm). RWUEP is the characteristic parameter of species. EP_0_ is crop water demand (mm); SWDF_2_ was the second water stress factor in the crop model.

#### 4.4.2. Crop Dry Matter Accumulation Equation

The formula for dry matter accumulation in the DSSAT model [38] is as follows:

(4) $$\Delta \mathrm{TOT}\;=\;0.758\;\times \left(\mathrm{PARCE}\;\times \;10^{-6}\;\times \;\mathrm{IPAR}-0.004\;\times \;\mathrm{TOT}\right)\;\times \;\mathrm{SWDF}$$

where ∆TOT is the increment of daily dry matter mass of crop (t/hm^2^); PARCE is photosynthetically active radiation conversion rate (g/MJ). IPAR is the amount of photosynthetically active radiation intercepted (MJ/hm^2^); TOT is the total dry matter (t/hm^2^); SWDF is a water stress factor affecting dry matter accumulation.

The PARCE calculation formula is as follows:

(5) $$\mathrm{PARCE}\;=\;{\mathrm{PARCE}}_{\max}\times \left\{1-\exp\left[-0.008\;\times \right.\right.\left.\left.\left(T-8\right)\right]\right\}$$

where PARCE_max_ is the maximum photosynthetically active radiation conversion efficiency (g/MJ); T is the average daily temperature (°C).

### 4.5. Model Calibration and Validation

The model parameters were calibrated against data for soil water content, LAI, and yield in 2022, whereas those for 2023 were used to validate the model. The Absolute Relative Error (ARE), Error Norm Root Mean Square (E_NRMS_), and the Coefficient of Determination (R^2^) [39,40,41] were used to assess the model performance for both the calibration and validation processes. It is widely acknowledged that a lower ARE, approaching 0, indicates higher accuracy. The value of E_NRMS_ under 10% is deemed exceptional; a range of 10% to 20% is good. When the value of R^2^ is close to 1, it indicated that the simulation accuracy of the model is acceptable.

(6) $$\mathrm{ARE}\;=\;\frac{\left|S_{i}-M_{i}\right|}{M_{i}}\times 100\%$$

(7) $$E_{\mathrm{NRMS}}=\frac{\sqrt{\frac{{\sum_{i=1}^{n}\left(M_{i}-S_{i}\right)}^{2}}{n}}}{M}\times 100\%$$

(8) $$R^{2}\;=\;1-\frac{\sum_{i=1}^{n}{\left(M_{i}-S_{i}\right)}^{2}}{\sum_{i=1}^{n}\left(M_{i}-M\right)}$$

where M_i_ is the measured value, S_i_ is the simulated value, M is the measured average value, S is the simulated average value, and n is the number of samples.

## 5. Conclusions

In this paper, Dssat-Forages-Alfalfa model was used to simulate the growth process of alfalfa under different irrigation quotas, and the effects of different initial soil water contents on yield before turning green were further studied. The main findings are summarized as follows:

(1) The simulation accuracy of the constructed model is acceptable. The Average Relative Error (ARE) is less than 10% and Normalized Root Mean Square Error (E_NRMS_) is less than 15%, while R^2^ is equal to or exceeding 0.85.
(2) Water consumption in the surface soil layer (0–20 cm) of the alfalfa was 6~12% and 13~31% more than that in the deeper soil layers of 20~40 cm and 40~60 cm, respectively. Leaf area in the second year was 7.90~10.32% larger than that in the first year. The yield of the second harvest was 800~1300 kg/ha greater than that of the first harvest.
(3) An irrigation scheme with an irrigation quota of 300 mm and irrigating ten times during the growth period was recommended. To ensure the maximization of alfalfa turning green rate, while ensuring efficient utilization of water resources and economic benefits, it was recommended to control the initial soil water content to 0.8 θ*_fc_*~1.0 θ*_fc_* and have an irrigation quota of 30 mm.

## Figures and Tables

**Table 1:** Evaluation of accuracy indicators for LAI and yield of alfalfa.

	Test Treatment	Statistical Index	ARE%	E_NRMS_	R^2^
2022 (Model calibration)	33 mm	LAI/(cm^2^·cm^−2^)	6.68	10.38	0.90
		Yield/(kg·hm^−2^)	5.87	10.74	0.89
	30 mm	LAI/(cm^2^·cm^−2^)	7.67	11.9	0.88
		Yield/(kg·hm^−2^)	4.49	9.91	0.91
	27 mm	LAI/(cm^2^·cm^−2^)	8.87	13.47	0.85
		Yield/(kg·hm^−2^)	5.53	10.26	0.90
	24 mm	LAI/(cm^2^·cm^−2^)	5.34	9.38	0.92
		Yield/(kg·hm^−2^)	3.77	9.26	0.93
2023 (Model validation)	33 mm	LAI/(cm^2^·cm^−2^)	7.46	11.63	0.88
		Yield/(kg·hm^−2^)	3.86	9.47	0.92
	30 mm	LAI/(cm^2^·cm^−2^)	8.11	12.88	0.87
		Yield/(kg·hm^−2^)	5.61	10.37	0.91
	27 mm	LAI/(cm^2^·cm^−2^)	6.52	10.27	0.91
		Yield/(kg·hm^−2^)	2.94	8.91	0.94
	24 mm	LAI/(cm^2^·cm^−2^)	7.97	12.11	0.88
		Yield/(kg·hm^−2^)	5.23	10.06	0.91

**Table 2:** Evaluation of accuracy indicators for soil water content.

	Test Treatment	Soil Depth/cm	ARE/%	E_NRMS_	R^2^
2022 (Model calibration)	33 mm	0~20	4.39	12.72	0.87
		20~40	2.26	9.42	0.92
		40~60	1.45	9.03	0.93
	30 mm	0~20	3.64	11.27	0.88
		20~40	1.92	9.87	0.92
		40~60	3.08	10.51	0.90
	27 mm	0~20	5.73	14.53	0.85
		20~40	4.67	10.64	0.89
		40~60	3.32	10.26	0.90
	24 mm	0~20	4.76	13.42	0.86
		20~40	3.10	11.09	0.89
		40~60	1.03	8.79	0.95
2023 (Model validation)	33 mm	0~20	5.04	13.73	0.87
		20~40	4.66	10.13	0.90
		40~60	2.38	9.66	0.92
	30 mm	0~20	4.12	12.24	0.88
		20~40	3.70	11.74	0.89
		40~60	1.99	9.94	0.92
	27 mm	0~20	4.06	12.08	0.89
		20~40	1.84	9.68	0.93
		40~60	1.01	8.49	0.95
	24 mm	0~20	4.87	13.34	0.87
		20~40	3.69	10.88	0.90
		40~60	1.63	9.46	0.93

**Table 3:** Calibrated values of genetic parameters of alfalfa varieties.

Argument	Definition	Calibrated Value
CSDL	Critical short day duration (h)	10.5
PPSEN	Relative response slope to photoperiod (1/h)	0.2
EM-FL	Duration of light and heat from seedling emergence to first blossom appearance (d)	21.5
FL-SH	From the initial inflorescence blossoming to the first inflorescence fruit setting, light, and heat conditions (d)	6.7
SD-PM	Photothermal duration from seed production to the first inflorescence’s physiological ripening (d)	33.5
FL-LF	The photothermal time between the flowering of the first inflorescence and the cessation of leaf expansion (d)	16
LFMAX	Maximum photosynthetic rate of leaves (mg CO_2_/m^2^·s^−1^)	2.4
SLAVR	Specific leaf area (cm^2^/g)	300
SIZLF	Maximum blade size (cm^2^)	5

**Table 4:** Irrigation scheduling for alfalfa from 2022 to 2023.

Harvest Stage	Growth Stage	Irrigation Date		Irrigation Quota/mm			
		2022	2023	W1	W2	W3	W4
First harvest	Turning green	16 May	15 May	24	27	30	33
	Branching	7 June	6 June	24	27	30	33
		20 June	22 June	24	27	30	33
	Budding	1 July	3 July	24	27	30	33
	Flowering	13 July	19 July	24	27	30	33
Second harvest	Turning green	30 July	4 August	24	27	30	33
	Branching	15 August	17 August	24	27	30	33
		27 August	29 August	24	27	30	33
	Budding	5 September	7 September	24	27	30	33
	Flowering	16 September	17 September	24	27	30	33
Total				240	270	300	330

**Table 5:** Fertilization program for alfalfa form 2022 to 2023.

Harvest Stage	Growth Stage	Fertilization Date		Fertilization Type	The Application Rate of Fertilizer/kg·hm^−2^	
		2022	2023		2022	2023
First harvest	Turning green	16 May	15 May	Urea	150	150
	Branching	20 June	22 June	Urea	150	150
Second harvest	Turning green	30 July	4 August	Urea	150	150
	Branching	27 August	29 August	Urea	150	150
Total					600	600

**Table 6:** Physical properties of soil at the test site.

Soil Depth	Clay%	Silt%	Sand%	Soil Bulk Density	Wilting Coefficient	Field Capacity	Saturated Water Content
(cm)	(<0.02 mm)	(0.02~0.5 mm)	(0.5~2 mm)	(g/cm^3^)	(cm^3^/cm^3^)	(cm^3^/cm^3^)	(cm^3^/cm^3^)
0–20	3.42	48.47	48.47	1.47	0.07	0.38	0.43
20–40	3.08	18.58	78.34	1.59	0.07	0.34	0.39
40–60	2.69	11.56	85.75	1.63	0.07	0.32	0.37
60–80	2.54	10.03	87.43	1.69	0.09	0.32	0.36
80–100	1.46	8.87	89.67	1.74	0.09	0.28	0.34

## Data Availability

Data are contained within the article.

## References

[1] Duan D., Zhao L., Zhao F. (2023). Application of alfalfa silage on cattle and sheep feed. Feed Res..

[2] Wang Q., Xie J., Yu L. (2023). Research Progress and Prospect of Alfalfa Breeding in China. J. Grassl. Forage Sci..

[3] Quan J., Li X., Gan H. (2023). Adaptability of 43 Medicago sativa varieties in the irrigated agricultural area of Zhangye, Gansu Province. Pratac. Sci..

[4] Duan Y., Tuo D., Zhao P. (2013). Crops caloric value and adjustment of planting structure in rainfed farmland of north Yinshan Mountian area, Inner Mongolia. J. Arid Land Resour. Environ..

[5] Kang W., Shi X., Duan H. (2023). Analysis of Water Requirement and Water Requirement Law of Main Crops in Dryland Area at The Northern Foot of Yinshan Mountain. J. Tianjin Agric. Sci..

[6] Zhang Y., Liang Z., Sui L. (2009). Effect on physiological characteristic of Medicago sativa under saline-alkali stress at seeding stage. Acta Pratac. Sin..

[7] Ma Y., Wang J., Wang C. (2023). Effects of water and fertilizer regulation on yield, quality and water and nitrogen use efficiency of alfalfa. Water Conserv. Plan. Des..

[8] Tian D., Hou C., Xu B. (2023). Combined Effect of Water and Salt Stress on Growth and Physiological Traits of Alfalfa at Branching Stage. J. Iirrigation Drain..

[9] Tong C., Li H., Niu H. (2019). TheImpact of Soil Moisture Control on Crop Coefficient and Water Use Efficiency of Drip-irrigated Alfalfa. Chin. Agric. Sci. Bull..

[10] Ma M., Zhao J., Li D. (2023). Effects of alflfa different irrigation methods on soil moisture and irrigation water use efficiency. Xinjiang Agric. Sci..

[11] Zheng H., Wang B., Cao X. (2023). Optimization of Subsurface Drip Irrigation Schedule of Alfalfa in Northwest China. Water.

[12] Wang X., Huang W., Yu S. (2019). Effect of water and fertilizer coupling on seed yield and composition of alfalfa grown with underground drip irrigation in Ningxia. Acta Pratac. Sin..

[13] Jones J.W., Hoogenboom G., Porter C.H. (2003). The DSSAT cropping system model. Eur. J. Agron..

[14] Abayechaw D. (2021). Review on decision support system for agrotechnology transfer (DSSAT) model. Int. J. Intell. Inf. Syst..

[15] Tan H., Lv X., Zhang Z. (2019). Influence of Climate Change on Cotton Production Potential Based on DSSAT Model Simulation. Cott. Sci..

[16] Wang Y. (2020). Simulation and Verification of Crop Growth Models with DifferentDrip Irrigation Models of Xinjiang Spring Wheat. Shihezi Univ..

[17] Ge J., Yu Z., Gong X. (2023). Evaluation of Irrigation Modes for Greenhouse Drip Irrigation Tomatoes Based on AquaCrop and DSSAT Models. Plants.

[18] Wang B., Yang H., Feng J. (2023). Rice Genotype Coefficient Optimization of DSSAT Based on PSO. Trans. Chin. Soc. Agric. Mach..

[19] Zhu B., Chen S., Xu Z. (2023). The Estimation of Maize Grain Protein Content and Yield by Assimilating LAI and LNA, Retrieved from Canopy Remote Sensing Data, into the DSSAT Model. Remote Sens..

[20] Wang Y., Jiang K., Shen H. (2023). Decision-making method for maize irrigation in supplementary irrigation areas based on the DSSAT model and a genetic algorithm. Agric. Water Manag..

[21] Song L. (2021). Simulation of Maize Growth and Yield under Climate Change in China.

[22] Xiang Z., Bailey R.T., Kisekka I. (2022). Using DSSAT-MODFLOW to determine the controls of groundwater storage and crop yield in groundwater-based irrigated regions. J. Hydrol..

[23] Shen H., Gao Y., Guo F. (2021). A modified DSSAT-CERES model for simulating summer maize growth under film mulching. Agron. J..

[24] Qu C., Li X., Hui J.U. (2019). The impacts of climate change on wheat yield in the Huang-Huai-Hai Plain of China using DSSAT-CERES-Wheat model under different climate scenarios. J. Integr. Agric..

[25] Xin M. (2022). Coupling Effect of Water and Nitrogen on Controlled Release Fertilizer Application and Optimization of Irrigation and Fertilization System in Salinized Irrigated Area.

[26] Wang X., Xin L., Du J. (2022). Simulation of Cotton Growth and Yield under Film Drip Irrigation Condition Based on DSSAT Model in Southern Xinjiang. Trans. Chin. Soc. Agric. Mach..

[27] Du J., Wang Z., Yu H. (2019). Optimum winter irrigation amount of subsurface drip irrigation in alfalfa grassland. Agric. Res. Arid Areas.

[28] Zhang S. (2018). Research on the Key Technical Parameters and Optimizingirrigation Schedule in Underground Drip Irrigation.

[29] Hou C. (2022). Effect Mechanism of Water and Salt Stress on Water and NitrogenUtilization and Physiological Characteristics of Alfalfa in Hetao Irrigation Area.

[30] Wu J., Miao S., Xu B. (2017). Distribution of Stable Hydrogen and Oxygen Isotopes in the Root Zone of Alfalfa under Drip Irrigation. J. Irrig. Drain..

[31] Tian D., Hou C., Xu B. (2019). Effect of Alfalfa on Water Consumption Process and Salt Changes in Fields. Trans. Chin. Soc. Agric. Mach..

[32] Meng F., Jin N., Wang Z. (2023). Effects of light and water on alfalfa growth and water use. Prata. L Sci..

[33] Tong C., Li H., Bai B. (2016). The Optimal Irrigation System of Alfalfa Based on WIN ISAREG Model. Chin. Agric. Sci. Bull..

[34] Wang X., Mi F., Li H. (2019). Comparison of yield, nutrition quality and winter surviving rate of alfalfa varieties with different fall dormancy levels. J. Northwest Sci-Tech Univ. Agric. For. Nat. Sci. Ed..

[35] Bao J. (2020). The Response of Alfalfa Planting and Harvesting Schemes to its Growth Periods.

[36] Zhu Y., Tang L., Liu L. (2019). Research Progress on the Crop Growth Model Crop Grow. Sci. Agric. Sin..

[37] Sarkar R. (2009). Use of DSSAT to model cropping systems. CABI Rev..

[38] Wang P. (2022). Research of Optimizing Deficit Irrigation for Sugarcane in Guangxi Province Using the DSSAT Model Based on Genetic Algorithm.

[39] Sun Y., Sun S., Li M. (2023). Study on high and stable yield area of soybean in Northeast China based on DSSAT model. Chin. J. Agric. Resour. Reg. Plan..

[40] Li Y., Shi P. (2020). Optimization of irrigation scheduling for spring wheat in Hetao Plain with DSSAT model. J. Nat. Sci. Beijing Norm. Univ..

[41] Yang X., Zheng L., Yang Q. (2018). Modelling the effects of conservation tillage on crop water productivity, soil water dynamics and evapotranspiration of a maize-winter wheat-soybean rotation system on the Loess Plateau of China using APSIM. Agric. Syst..

